# The Vulnerability to Suicidal Behavior is Associated with Reduced Connectivity Strength

**DOI:** 10.3389/fnhum.2015.00632

**Published:** 2015-11-30

**Authors:** Stijn Bijttebier, Karen Caeyenberghs, Hans van den Ameele, Eric Achten, Dan Rujescu, Koen Titeca, Cornelis van Heeringen

**Affiliations:** ^1^Unit for Suicide Research, Department of Psychiatry and Medical Psychology, Ghent UniversityGhent, Belgium; ^2^School of Psychology, Faculty of Health Sciences, Australian Catholic UniversityMelbourne, VIC, Australia; ^3^Department of Psychiatry, AZ Sint-Jan Brugge-Oostende AVBrugge, Belgium; ^4^Department of Radiology and Nuclear Medicine, Ghent UniversityGhent, Belgium; ^5^Ghent Institute for Functional and Metabolic Imaging (GIfMI), Ghent UniversityGhent, Belgium; ^6^Universitätsklinik und Poliklinik für Psychiatrie, Psychotherapie und Psychosomatik, Martin-Luther-Universität Halle-WittenbergHalle/Saale, Germany; ^7^Department of Psychiatry, AZ GroeningeKortrijk, Belgium

**Keywords:** suicidal behavior, predisposition, connectivity strength, network based analysis, 5-HTTLPR, MAOA, whole brain tractography

## Abstract

Suicidal behavior constitutes a major public health problem. Based on the stress–diathesis model, biological correlates of a diathesis might help to predict risk after stressor-exposure. Structural changes in cortical and subcortical areas and their connections have increasingly been linked with the diathesis. The current study identified structural network changes associated with a diathesis using a whole-brain approach by examining the structural connectivity between regions in euthymic suicide attempters (SA). In addition, the association between connectivity measures, clinical and genetic characteristics was investigated. We hypothesized that SA showed lower connectivity strength, associated with an increased severity of general clinical characteristics and an elevated expression of short alleles in serotonin polymorphisms. Thirteen euthymic SA were compared with fifteen euthymic non-attempters and seventeen healthy controls (HC). Clinical characteristics and three serotonin-related genetic polymorphisms were assessed. Diffusion MRI together with anatomical scans were administered. Preprocessing was performed using Explore DTI. Whole brain tractography of the diffusion-weighted images was followed by a number of streamlines-weighted network analysis using NBS. The network analysis revealed decreased connectivity strength in SA in the connections between the left olfactory cortex and left anterior cingulate gyrus. Furthermore, SA had increased suicidal ideation, hopelessness and self-reported depression, but did not show any differences for the genetic polymorphisms. Finally, lower connectivity strength between the right calcarine fissure and the left middle occipital gyrus was associated with increased trait anxiety severity (*r_s_* = −0.78, *p* < 0.01) and hopelessness (*r_s_* = −0.76, *p* < 0.01). SA showed differences in white matter network connectivity strength associated with clinical characteristics. Together, these variables could play an important role in predicting suicidal behavior.

## Introduction

Suicidal behavior constitutes a major public health problem. It is estimated that more than 800,000 individuals worldwide take their own lives annually, while numbers of non-fatal suicidal behavior are 10–20 times higher (WHO, 2014). Prediction of risk of suicide and identification of treatment and prevention targets beyond major psychiatric illnesses are objectives to improve suicide prevention. Available clinical predictors are scarce and no biomarkers have been established yet to help clinicians to predict suicidal behavior or to target with treatment. A proposed stress—diathesis model describes suicidal behavior as the result of an interaction between stressors and a predisposition or diathesis to suicidal behavior (Hawton and van Heeringen, 2009). Most people with major psychiatric disorders never manifest suicidal behavior, indicating the importance of the diathesis in addition to the disorder. The biological correlates of the diathesis could provide predictors for suicide risk that are distinguishable from vulnerabilities of co-occurring psychiatric disorders and that might help to predict risk after exposure to stressors such as an acute psychiatric disorder or adverse psychosocial events (van Heeringen and Mann, 2014). Investigating the association with genetic polymorphisms could furthermore show how these vulnerabilities relate to known suicidal behavior genes.

*In vivo* brain imaging has been used increasingly in the last decade to study the diathesis, and a number of reviews and a meta-analysis have revealed an association between functional and structural changes in cortical brain regions and a diathesis (van Heeringen et al., 2011; Desmyter et al., 2013; van Heeringen et al., 2014). A number of studies have recently suggested the involvement of structural white matter changes, e.g., increased fractional anisotropy (FA) and a reduction in fiber projection, in the connectivity between brain regions in the diathesis (Lo et al., 2007; Jia et al., 2010, 2014; Yurgelun-Todd et al., 2011; Mahon et al., 2012; Lopez-Larson et al., 2013; Olvet et al., 2014; Kim et al., 2015). For example, Jia et al. (2014) found reduced fiber projections for suicide attempters (SA) in the frontothalamic loops passing through the anterior limb of the internal capsule. However, most of these studies used a region-of-interest approach rather than an unbiased whole-brain analysis of connectivity and none of the studies examined the strength of the connections using a network analysis. Moreover, study populations often comprised non-euthymic subjects, being acutely depressed.

Network analysis may provide more insights into structural changes, which may be too subtle to be detected at the local level. We therefore investigated structural network connectivity strength using an explorative network-based diffusion Magnetic Resonance Imaging (MRI) analysis. Structural connectivity strength can be seen as the strength of the structural WM connection between two cortical areas. Streamline count was defined as a weight, which represents the number of fiber tracts connecting two distinct brain regions. This weight has previously been used as a proxy marker of structural connectivity (Skudlarski et al., 2008; Jones et al., 2013; Khalsa et al., 2014). In order to compare the constructed networks, we made use of the Network Based Statistics (NBS) toolbox (Zalesky et al., 2010). NBS uses nonparametric statistics to deal with the multiple comparison problem of a graph. A graph is a mathematical representation of a network, with regional cortical areas, named nodes, being interconnected by interregional anatomical white matter connections, named edges. The magnitude of an edge can be quantified as the strength of connectivity. NBS thus allows us to quantify network properties and to define brain networks.

NBS has been previously used to map structural networks in psychiatric disorders including schizophrenia, depression and mild cognitive impairment (Fornito et al., 2011; Zalesky et al., 2011; Bai et al., 2012). For example, Zalesky et al. (2011) found impaired connectivity in white matter architecture in a network encompassing the medial frontal, parietal, occipital and left temporal cortex, with these regions showing less efficient interconnectivity, in schizophrenics.

As far as known to the authors, connectivity strength has not yet been studied in SA. Moreover, in the present study we included only euthymic individuals in order to assess whether findings are associated with the diathesis and not with state-dependent characteristics, such as depression. The findings could thus be translate to a general, currently non-clinical population, increasing targeted preventative actions for at-risk groups and individuals.

Finally, another goal of the present study was to investigate the association between structural connectivity and polymorphisms of candidate genes such as the Serotonin transporter gene promoter (*5-HTTLPR*) variable number tandem repeat (*VNTR*) [*rs4795541 (L/S-variant)* and *rs25531* (*L_A_/L_G_/S-variant*)] and Monoamine oxidase A (*MAOA*) polymorphisms. These polymorphisms have been shown to interact with stressful life events, increasing the risk of suicidal behavior (Antypa et al., 2013).

Based on previous studies investigating white matter differences in SA, we hypothesized that patients who have a history of suicidal behavior showed lower connectivity strength, associated with an increased severity of general clinical characteristics and an elevated expression of short alleles in serotonin polymorphisms. Specifically, connectivity strength is hypothesized to be decreased between prefrontal cortical regions and limbic regions.

## Materials and Methods

### Participants

Thirteen euthymic outpatients with a history of suicide attempts (SAs) and 15 euthymic outpatients without a history of suicide attempt (NA) were included. All patients had a history of depression. They were recruited in different centers in the Flemish part of Belgium.

Twenty-two healthy controls (HC), without any history of depression or suicidal behavior, were included.

Only right-handed participants aged between 18 and 65 years old with their four grandparents originating from Western Europe were included. Subjects were screened for current depressive symptoms and general psychopathology using the Hamilton depression rating scale (Hamilton, 1960) and the Dutch version of the MINI International Neuropsychiatric Interview (Overbeek et al., 1999), respectively. Subjects needed to have a maximum score of 7 on the Hamilton depression rating scale (Zimmerman et al., 2013). The MINI was used to rule out lifetime bipolar disorder, lifetime psychotic disorder and alcohol abuse in the past 12 months, as these were exclusion criteria. Possible neurological disorders and current medication use were questioned in detail by the primary researcher. Subjects with a lifetime history of neurological disorders or using psychotropic medication at time of participation, except for selective serotonin re-uptake inhibitors and serotonin–norepinephrine reuptake inhibitors, were excluded.

The study was approved by the Ghent University Hospital ethical comity. Written informed consents were obtained from each participant.

### Clinical Assessment

First, we assessed characteristics of plans and wishes to commit suicide for all participants using the Scale for Suicidal Ideation (Beck et al., 1979). Each item consists of three options graded according to suicidal intensity ranging from 0–2. The total score is yielded by the sum of the ratings for the first 19 items, ranging from 0–38. The Beck Hopelessness Scale was then administered to examine an individual’s thoughts and beliefs about the future (Beck et al., 1974; Beck and Steer, 1988). This scale comprises 20 items which participants have to answer with correct or incorrect, with a maximum score of twenty.

Thirdly, self-reported impulsivity was measured using the Barratt impulsiveness scale (Patton et al., 1995), followed by an assessment of state and trait anxiety using the Dutch version of the State-Trait Anxiety Inventory, the Zelf-beoordelings vragenlijst (van der Ploeg, 2000).

Fourthly, self-reported depressive symptoms were assessed using the Dutch version of the Beck depression inventory (BDI-II-NL; Beck et al., 1961).

The Dutch version of the BIS/BAS Scale was then administered to assess behavioral approach and inhibition, and is subdivided in four categories: Fun Seeking (BAS-Fun), Reward Responsiveness (BAS-Reward), Drive (BAS-Drive) and concerns regarding the possible occurrence of negative events (Franken et al., 2005). The drive scale measured the level of persistence in pursuit of desired goals, while the fun seeking scale reflected a desire for rewards and the willingness to impulsively approach a potentially rewarding event. The reward responsiveness scale on the other hand focused on positive reactions in anticipation of reward.

Finally, the Dutch version of the Structured Clinical Interview for DSM-IV Axis II Personality Disorders was used to assess the presence of personality disorder(s) (First et al., 1994).

### DNA Samples

Saliva was collected using a self-collection kit following the instructions of the manufacturer (Oragene DNA Self-Collection Kit (OG-500); DNA Genotek, Inc., Ontario, Canada). We focused on three polymorphisms, namely *MAOA uVNTR* and two variants of *SLC6A4 5-HTTLPR*, *rs4795541 (L/S-variant)* and *rs25531* (*L_A_/L_G_/S-variant*) which will be described in the next section.

#### SLC6A4 5-HTTLPR: rs4795541 (L/S-variant) and rs25531 (*L_A_/L_G_/S-variant*)

The primer sequences used for the *5-HTTLPR VNTR*, *rs4795541 (L/S-variant)*, were *5′-CAACTCCCTGTACCCCTCCTA-3′* (forward) and *5′-GGTTGCAGGGGAGATCCTG-3′* (reverse), resulting in a 147 base pairs (bp) (short allele S) and a 190 bp PCR product (long allele L). The reaction mixture contained 5 ng genomic DNA, 2.5 mM MgCl_2_, 60 mM Tris-HCl, 15 mM ammonium sulfate, 5 μl Q-solution (Qiagen, Venlo, the Netherlands), 6.7 nmol forward and 4.4 nmol reverse primer, 40 nmol dNTP, and 1 U Taq polymerase (Fermentas, Waltham, MA, USA). The temperature profile consisted of an initial denaturation at 94°C for 5 min, followed by 45 cycles of 30 s at 94°C denaturation, 30 s at 56.8°C annealing, and 60 s at 72°C elongation, followed by final elongation for 7 min at 72°C. Ten microliter of the PCR products were separated on a 2% agarose gel at 100 V for 45 min.

For *5-HTTLPR VNTR, rs25531* (*L_A_/L_G_/S variants*) 15 μl of the above PCR reaction were digested with 2 U MspI (Fermentas, Waltham, MA, USA) which results in 147 bp S, 190 bp for L_A_ and 83 bp + 107 bp for L_G_ variants. Fragments were separated on a 2% agarose gel (100 V, 45 min) stained with ethidium bromide.

#### MAOA uVNTR

The primer sequences used for the *MAOA VNTR* were *5′-TGCTCCAGAAACATGAGCAC-3′* (forward) and *5′-ATTGGGGAGTGTATGCTGGA-3′* (reverse), resulting in 350 bp (2R), 380 bp (3R), 395 bp (3.5R), 410 bp (4R) and 440 bp (5R) PCR products. The reaction mixture contained 5 ng genomic DNA, 2.5 mM MgCl_2_, 60 mM Tris-HCl, 15 mM ammonium sulfate, 5 nmol of each primer, 40 nmol dNTP, and 1U Taq polymerase (Fermentas, Waltham, MA, USA). The temperature profile consisted of an initial denaturation at 94°C for 5 min, followed by 35 cycles of 30 s at 94°C denaturation, 30 s at 56°C annealing, and 60 s at 72°C elongation, followed by final elongation for 10 min at 72°C. 15 μl of the PCR products were separated on a 2.5% agarose gel at 100 V for 55 min.

### MRI Data Acquisition

A Siemens 3T Magnetom Trio Tim MRI scanner (Siemens, Erlangen, Germany) with an 32-channel standard head coil was used for image acquisition.

High resolution T1-weighted structural images were acquired using a three-dimension magnetization prepared rapid gradient-echo [MPRAGE; *repetition time* (TR) = 1550 ms, *echo time* (TE) = 2.39 ms, 0.9 × 0.9 × 0.9 mm^3^ voxel size, field of view 192 × 192 mm^2^, 176 sagittal slices, acquisition time of 5 min and 56 s] for anatomical detail, echo planar imaging (EPI) correction and co-registration during the diffusion MRI image processing.

Three sets of diffusion-weighted images (single-shot spin-echo) were acquired with slice *thickness* = 2.0 mm, *TR* = 7700 ms, *TE* = 83 ms, number of sagittal *slices* = 60, voxel *size* = 2.0 × 2.0 × 2.0 mm^3^ and acquisition time of 6 min and 23 s. Implemented b values were 0, and 850 s/mm^2^, applied in 12 uniformly distributed directions. Each set contained 12 volumes and one reference image, providing a total of 39 volumes.

### Image Processing

#### Preprocessing

ExploreDTI, version 4.8.4, was used to preprocess the diffusion data (Leemans et al., 2009). The following steps were taken: (1) All diffusion-weighted imaging volumes (DWI’s) were visually inspected to detect apparent artifacts in the data, such as large signal dropouts and geometric distortions. The images were inspected from different “orthogonal” views to detect any interslice and intravolume instabilities (Jones and Leemans, 2011). Data was further checked by assessing the average residuals across DWI’s and inspecting the outlier profiles for each subject. (2) The diffusion-weighted datasets were corrected for subject motion and eddy current-induced geometrical distortions, while co-registering the FreeSurfer-converted T1 MRI images for reduction of EPI susceptibility distortions. The B-matrix was reoriented appropriately during motion correction (Leemans and Jones, 2009). Following this step, we re-assessed the data quality using the same procedure as described in step 1, also checking whether head movement did not exceed the voxel size (i.e., 2 mm) for each plane and if co-registration with the FreeSurfer-converted T1-images was satisfactory. Five subjects, all HC, were excluded from analysis due to excessive movement artifacts during the MRI scans.

#### Tractography

A whole brain tractography was performed for the 45 remaining subjects in ExploreDTI, version 4.8.4, using a deterministic streamline approach (Basser et al., 2000b; Leemans et al., 2009; Jeurissen et al., 2011), with a uniform seed point resolution of 2 mm^3^, and an fiber tract voxel FA value of < 0.2 or an angle threshold of 30 degrees (Hagmann et al., 2008; Gong et al., 2009; Caeyenberghs et al., 2015).

##### Nodes and edge weights

**Figure 1:**
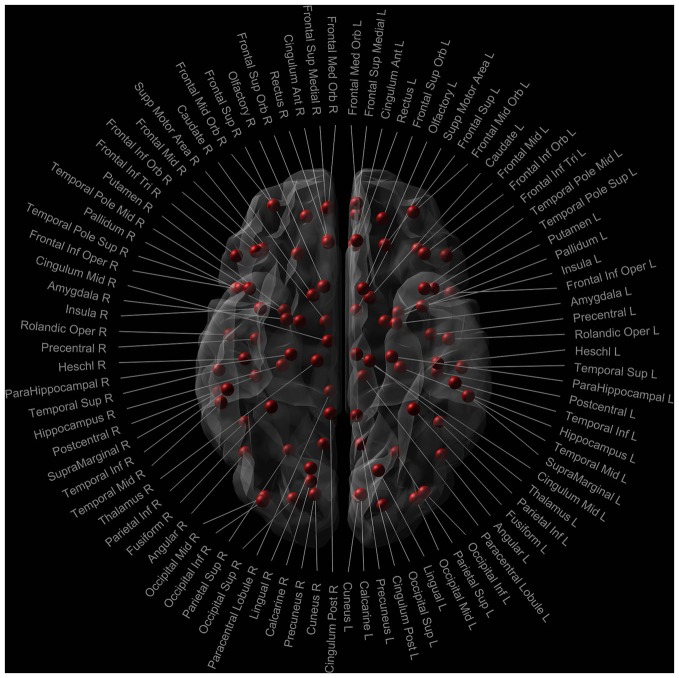
**Nodes of the AAL template used in the network analysis.** Red dots signify the 90 cerebral nodes. Each node is labelled with the anatomical name used in the AAL template.

The whole brain tracts were parcellated using the Automated Anatomic Labeling (AAL) template (Tzourio-Mazoyer et al., 2002). In this study, we only included the 90 cerebral brain regions (excluding the cerebellum) to obtain the connectivity matrices for each subject (Figure 1). Number of streamlines was computed as weight.

##### NBS analysis of the structural network

The obtained weighted matrices of the previous step were concatenated and imported in the NBS toolbox. Our data was analyzed using the default parameters from the NBS Toolbox (Zalesky et al., 2010), as applied in previous studies (Poudel et al., 2014; Caeyenberghs et al., 2015). Specifically, NBS was used to identify pairs of regions whereby the structural connectivity strength was altered. A one-way analysis of variance was performed to identify the overall group effect (F-contrast). Pairs of regions were withheld using the highest possible F-statistic (*F* > 3.5). These pairs got a family-wise-error corrected *p*-value (*p* < 0.05) ascribed using permutation tests. A total of 5000 permutations were generated, assigning participants randomly to one of the three groups for each permutation. Then, connectivity strengths was extracted for all significant pairs of regions, and further analyzed in a statistical package (IBM SPSS Statistics 21, Armonk, NY, USA). Finally, non-parametric *Mann*-*Whitney U tests* were employed to further disentangle the significant F-effect and identify networks which differed between the groups, using the following planned *post hoc* contrasts: HC and NA, HC and SA and NA and SA.

### Statistical Analyses

Non-parametric *Kruskal-Wallis tests* were conducted on the clinical variables. Significant group effects were explored further using *Mann-Whitney U tests*. Categorical variables were analyzed with *Fisher’s Exact Tests*, as the condition for use of the chi-square analyses (each cell ≥ five observations) was not fulfilled.

An effect of 5-HTTLPR and MAOA polymorphisms was assessed by means of *post hoc Mann-Whitney U tests* following a *Kruskal-Wallis test* used to assess group differences.

To determine whether the connectivity strength in SA is correlated with the clinical and genotypic variables, Spearman’s rank correlation coefficient were calculated, using a Monte-Carlo-based permutation test with 100,000 permutations to correct for multiple comparisons. Due to the number of correlations examined, only correlations below a statistical significance level of *p* < 0.01 were considered significant (Caeyenberghs et al., 2012a,b).

## Results

### Demographic Characteristics

As groups were *a priori* matched for age, education level (completed years of education, starting from 6 years old) and IQ (Nederlandse leestest voor volwassenen; Schmand et al., 1992), non-parametric Kruskal Wallis tests revealed no significant differences between the three groups for age, education level and IQ. Only the gender ratio differed significantly between the HC and NA group (*p* < 0.05), but not between these groups and SA (Table 1). As gender ratio did not differ between SA and the other groups, this variable was not taken into account as covariate.

**Table 1:** **Demographic characteristics and clinical differences**.

Variable	HC^a^ (*n* = 17)	NA^a^ (*n* = 15)	SA^a^ (*n* = 13)	*p*-value^b^	*Post hoc* contrast^c^
**Demographic variables**
Age, years	26 (24)	36 (20)	31 (21)	0.45	
Education, years	15 (3)	15 (3)	15 (3)	0.35	
IQ, NLV score	101 (13)	106 (23)	105 (11)	0.79	
Female (%)	94	53	69	<0.05	HC > NA
**Clinical variables**
Suicide ideation, SSI score	0 (0)	0 (0)	0 (1)	<0.05	HC < SA
Hopelessness, BHS-score	2 (1)	3 (2)	3 (3)	< 0.05	HC < SA
Self-reported depression, BDI-score	1 (3)	3 (5)	5 (15)	<0.01	HC < SA
Stait Anxiety, ZBV score	29 (7)	34 (13)	32 (17)	0.08	
Trait Anxiety, ZBV score	29 (10)	36 (17)	45 (14)	< 0.01	HC <NA, SA
Fun Seeking, BAS score	12 (4)	10 (3)	11 (1)	<0.05	HC >NA, SA
Reward Responsiveness, BAS-score	18 (4)	17 (6)	18 (6)	0.70	
Drive, BAS score	13 (4)	11 (2)	12 (6)	<0.05	HC > NA
Avoidance, BIS score	21 (5)	24 (9)	20 (13)	0.28	
Impulsivity, BIS-11 score	57 (11)	50 (19)	59 (23)	0.40	
History of Axis II Diagnoses, DSM-IV (%)	0	0	8	0.29	
Medication use, SSRI and SNRI (%)	0	27	46	<0.01	HC < NA, SA

*^a^Except where indicated otherwise, values are the median and the interquartile range; ^b^Kruskal-Wallis tests for comparisons with continuous variables; Fisher’s Exact Tests for categorical variables; ^c^Mann-Whitney U test for continuous variables. Only significant post hoc tests are shown HC, Healthy Controls; L, Left; NA, Euthymic non-attempters; R, Right; SA, Euthymic suicide attempters*.

### Clinical Variables

As shown in Table 1, significant group differences were found for suicidal ideation (Scale for Suicidal Ideation), hopelessness (Beck Hopelessness Scale), subjective interpretation of current depression (BDI-II-NL), trait anxiety (zelf-beoordelings vragenlijst), and behavioral approach (fun seeking and drive; BIS/BAS scale). *Post hoc* test showed increased levels of hopelessness (Beck Hopelessness Scale), suicidal ideation (Scale for Suicidal Ideation) and self-reported depressive symptoms (BDI-II-NL) in SA, compared to HC, but not compared to NA. Behavioral approach (fun seeking and drive; BIS/BAS scale) was also increased in HC, compared to NA. HC and SA did not differ for the Drive scale. The fun seeking scale did show a significant difference between HC and SA, but not between SA and NA. There was no significant difference in trait anxiety between SA and NA. Both groups did, however, show significantly increased levels of trait anxiety when compared to HC.

### Network Differences

**Figure 2:**
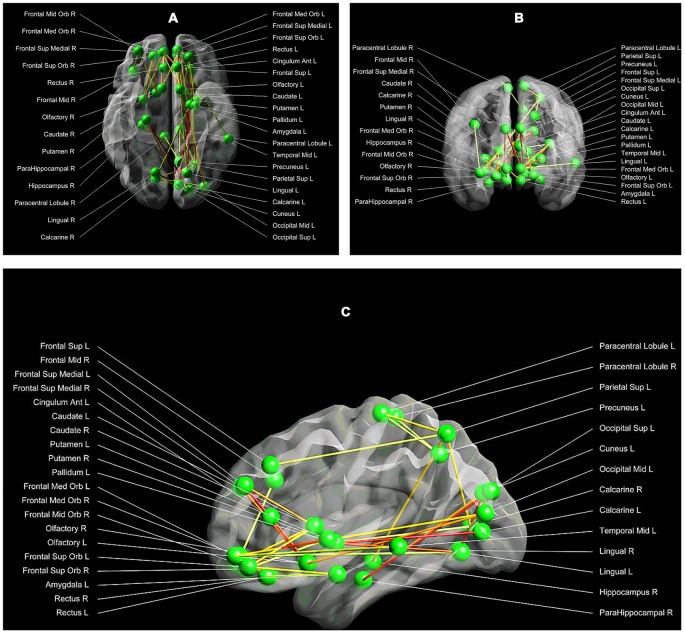
**(A–C)** Between group structural connectivity alterations. Respectively the axial **(A)**, coronal **(B)** and sagittal **(C)** view of the subnetwork, which demonstrated significant alterations in structural connectivity between the three groups. This network comprises 34 nodes and 36 edges. Each node is labelled with the anatomical name used in the AAL template. The lines represent the edges of the observed network, while the green dots symbolize the network nodes. The darker the line, the higher the strength of the network connection between a pair of nodes (with red being the highest connectivity strength, and white the lowest connectivity strength). Visualization: Explore DTI, version 4.8.4 (Leemans et al., 2009).

The NBS method identified one subnetwork (*p* < 0.05 family-wise-error corrected), consisting of 34 nodes and 36 connections, with significant alterations in structural connectivity between the three groups. This subnetwork encompassed both cortical, as well as subcortical regions (such as the amygdala, the anterior cingulum, the caudate and putamen) (*F* = 4.33, *p* < 0.05; Figures 2A–C).

**Figure 3:**
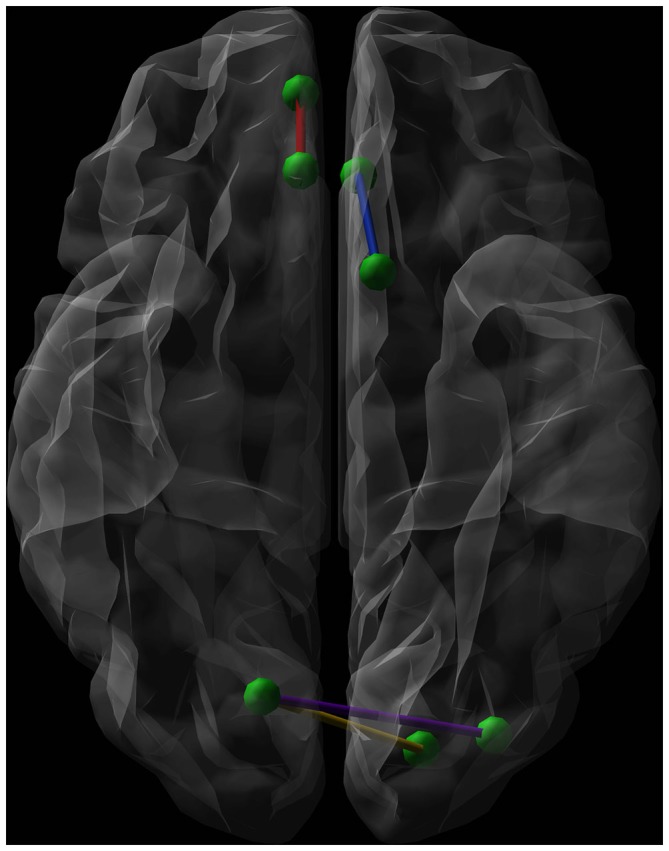
**Graphical representation (axial view, radiological convention) of the network connections showing a significantly lower connectivity strength when comparing euthymic suicide attempters (SA) with euthymic non-attempters and healthy controls (HC).** Green dots represent nodes. The blue line shows the connection between the left olfactory cortex and the left anterior cingulate gyrus, while the red line symbolizes the connection between the right medial orbital superior frontal gyrus and the right rectal gyrus. The purple line connects the right calcarine fissure and the left middle occipital gyrus. The yellow line forms the connection between the right calcarine fissure and both the left superior occipital gyrus.

As shown in Table 2, *post hoc* tests revealed that SA, compared to HC and NA, showed decreased connectivity strength between the left olfactory cortex and the left anterior cingulate gyrus (Connection 1). There was a trend towards significance (*p* = 0.07) for decreased connectivity strength in SA between the right medial orbital superior frontal gyrus and the right rectal gyrus (Connection 2), and between the right calcarine fissure and both the left superior and middle occipital gyrus (Connections 3 and 4 respectively). HC and NA did not differ significantly for each of these connections (Figure 3).

**Table 2:** **Connections involved in the vulnerability to suicidal behavior**.

Number of streamline	Connections	MNI Coordinates (mm) (*x, y, z*)	*Post hoc* contrasts (Mann–Whitney *U* tests)
	**Connection 1:**	(−8.06, 15.05, −12.00)	HC = NA (*U* = 123.0, *p* = 0.87);
	L olfactory cortex →	→	HC > SA (*U* = 38.5, *p* < 0.01);
	L anterior cingulate gyrus	(−4.04, 35.40, 13.95)	NA > SA (*U* = 41.0, *p* < 0.01)
	**Connection 2:**	(8.16, 51.67, −7.13)	HC = NA (*U* = 116.5, *p* = 0.68);
	R superior frontal gyrus,	→	HC > SA (*U* = 44.0, *p* < 0.01);
	medial orbital →	(8.35, 35.64, −18.04)	NA > SA (*U* = 58.0, *p* = 0. 07)
	R gyrus rectus
	**Connection 3:**	(15.99, −73.15, 9.40)	HC = NA (*U* = 89.0, *p* = 0.15);
	R calcarine fissure →	→	HC > SA (*U* = 46.0, *p* < 0.01);
	L superior occipital gyrus	(−16.54, −84.26, 28.17)	NA > SA (*U* = 59.0, *p* = 0.08)
	**Connection 4:**	(15.99, −73.15, 9.40)	HC = NA (*U* = 91.0, *p* = 0.17);
	R calcarine fissure →	→	HC > SA (*U* = 43.5, *p* < 0.01);
	L middle occipital gyrus	(−32.39, −80.73, 16.11)	NA > SA (*U* = 58.0, *p* = 0.07)

*HC, Healthy Controls; L, Left; NA, Euthymic non-attempters; R, Right; SA, Euthymic suicide attempters*.

For the remaining 32 contrasts, *post hoc* tests did not show a significant difference between SA compared with NA and HC. These results are presented in the Supplementary Table 1.

### Genotypic Differences

Fisher’s Exact Tests revealed a significant group difference for the *MAOA-uVNTR* polymorphism (*p* < 0.01), with HC differing significantly from NA (*p* < 0.01) and SA (*p* < 0.05). No significant differences were found between NA and SA (*p* = 0.24).

No significant differences were observed between the groups for the *5-HTTLPR_VNTR* polymorphism (*p* = 0.79) and *5-HTTLPR_rs25531* polymorphism (*p* = 0.89).

### Correlations

#### Total Group

**Figure 4:**
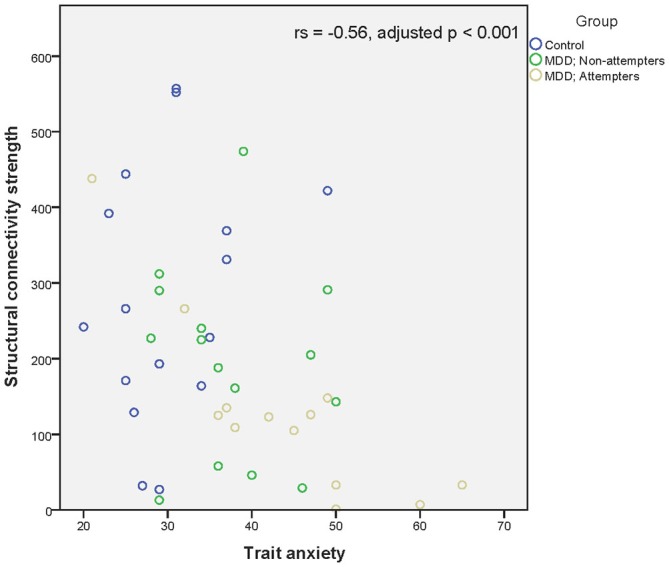
**Scatter plot of the significant association between the right calcarine fissure—left middle occipital gyrus structural connectivity strength and trait anxiety as measured by the Zelf-beoordelings vragenlijst.** A higher structural connectivity strength represents a higher number of streamlines for that subject. A higher score on the Zelf-beoordelings vragenlijst represents a higher level of trait anxiety. Associations were measured using the Spearman’s rank correlation coefficient.

**Figure 5:**
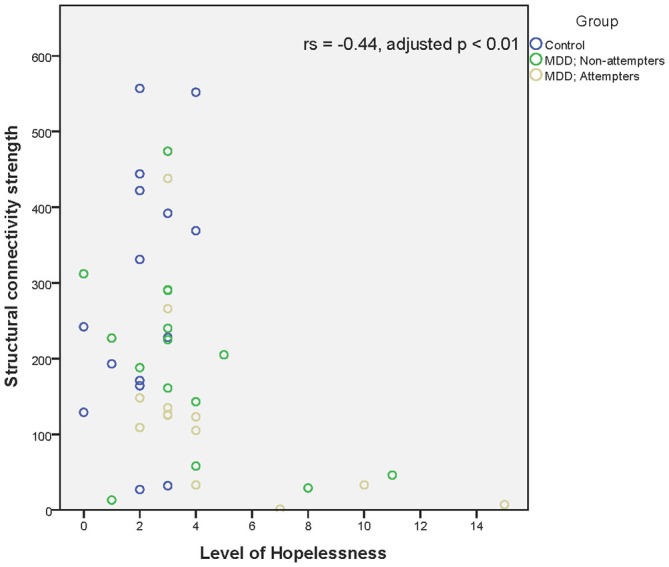
**Scatter plot of the significant association between the right calcarine fissure—left middle occipital gyrus structural connectivity strength and hopelessness as measured by the Beck Hopelessness scale.** A higher structural connectivity strength represents a higher number of streamlines for that subject. A higher score on the Beck Hopelessness Scale represents a higher level of hopelessness. Associations were measured using the Spearman’s rank correlation coefficient.

**Figure 6:**
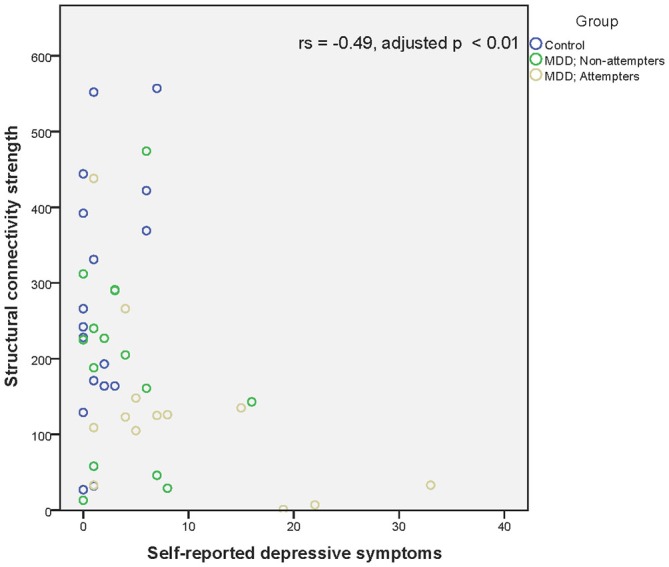
**Scatter plot of the significant association between the right calcarine fissure—left middle occipital gyrus structural connectivity strength and self-reported depressive symptoms as measured by the BDI-II-NL.** A higher structural connectivity strength represents a higher number of streamlines for that subject. A higher score on the BDI-II-NL represents a higher level of self-reported depressive symptoms. Associations were measured using the Spearman’s rank correlation coefficient.

Regarding associations between connectivity strength differences discriminating SA from NA and HC, and the clinical variables, there was a significant negative correlation between Connection 4 and trait anxiety (*r_s_* = −0.56, adjusted *p* < 0.001), hopelessness (*r_s_* = −0.44, adjusted *p* < 0.01), and self-reported depressive symptoms (*r_s_* = −0.49, adjusted *p* < 0.01), as can be seen in Figures 4–6. In other words, decreased structural connectivity between the right calcarine fissure and the left middle occipital gyrus was associated with increased severity of trait anxiety, hopelessness and subjective depressive symptoms.

#### Within Group Differences

SA showed significant associations between Connection 4 and both hopelessness (*r_s_* = −0.76, *p* < 0.01) and trait anxiety (*r_s_* = −0.78, *p* < 0.01). Hence, lower structural connectivity was associated with an increase in trait anxiety and hopelessness. Non-attempters did not show any significant association. Finally, no correlations between connectivity strength and genotype differences could be demonstrated.

## Discussion

As far as known to the authors this is the first comparative study of structural connectivity strength of networks in currently euthymic suicide attempters. The findings suggest changes in connectivity strength in association with a diathesis to suicidal behavior. Specifically, suicide attempters have a reduced strength in connectivity in frontal and occipital regions, ranging across both hemispheres.

Euthymic suicide attempters have increased levels of suicidal ideation, hopelessness and current subjective interpretation of depression, while genetic results do not reveal any significant differences in serotonin related polymorphisms in euthymic suicide attempters.

Furthermore, lower connectivity strength between the right calcarine fissure and the left middle occipital gyrus was associated with higher levels of trait anxiety, hopelessness and self-reported depressive symptoms. In suicide attempters alone, decreased connectivity strength between these two regions was associated only with a higher trait anxiety and hopelessness.

Preceding a discussion of these findings, a few methodological issues need to be addressed.

Our findings may have been affected by the drug treatment in some patients (Germana et al., 2010; Vernon et al., 2012). However, in the current study we have only included patients which used selective serotonin re-uptake inhibitors and serotonin–norepinephrine reuptake inhibitors, as the effect of these drugs on structural connectivity appears to be limited (Fan et al., 2012).

Second, sample size is limited due to strict inclusion criteria. Regardless, we were able to detect differences in connectivity strength between attempters and non-attempter groups. Nonetheless, group differences need to be reproduced in larger samples.

Thirdly, the acquisition of the diffusion-weighted images using 12 uniformly distributed directions could give less qualitative results when used for tractography when compared to sequences such as High Angular Resolution Diffusion-weighted Imaging. Although, studies have shown that data obtained using this sequence does provide qualitative results (Yamamoto et al., 2007; Lebel et al., 2012). Nonetheless, results should be interpreted with caution.

Fourthly, although number of streamlines is seen as the main weight for calculating structural connectivity strength (Rubinov and Bassett, 2011; Zalesky et al., 2011), by using only this measure as a weight to construct the connectivity matrices, we may have misinterpreted or missed certain results. Specifically, we were not able to correct for the individual volume of each node within the AAL template, nor were we able to compare the findings with other weights, such as FA. As this could potentially bias results, future research should take this in to account.

Finally, the edges of the structural network were defined using a deterministic tractography approach (Mori et al., 1999; Basser et al., 2000a). Previous studies showed that this method is not able to resolve crossing fiber bundles (Mori and van Zijl, 2002; Tournier et al., 2011; Jones et al., 2013). Thus, using this technique in a tractography can result in the loss of edges due to the elimination of fiber bundles. Recent studies have proposed tractography approaches based on more advanced diffusion models (Hess et al., 2006; Wedeen et al., 2008; Jeurissen et al., 2011), which may solve the fiber crossing problem by providing more accurate anatomical connectivity patterns of brain networks. These approaches should be taken in to account in future studies.

Results show that the lower connectivity strength, which differs between suicide attempters and non-attempters (HC and NA), is part of the corpus callosum. Specifically, the connection strength between the left olfactory cortex and the left anterior cingulate gyrus and between the medial orbital part of the right superior frontal gyrus and the right gyrus rectus corresponds with parts of the forceps minor, each in a different hemisphere, which connects the frontal lobes via the genu of the corpus callosum (Catani and Thiebaut de Schotten, 2008). Furthermore, the connection strengths between the right calcarine fissure and the left superior occipital gyrus, and between the right calcarine fissure and the left middle occipital gyrus appear to be parts of the forceps major (Catani and Thiebaut de Schotten, 2008). This tract interconnects the occipital lobes and runs through the splenium (Dougherty et al., 2005).

Previous research in suicide attempters has revealed involvement of parts of the corpus callosum in the diathesis to suicidal behavior, specifically structural characteristics of the genu and splenium (Cyprien et al., 2011; Kim et al., 2015), providing additional evidence for our results.

As the corpus callosum plays a vital role in interhemispheric interaction (Berlucchi et al., 1967; Ptito et al., 1981), the transfer of information between the left and right frontal and occipital lobe appears to be hampered in suicide attempters due to a decreased connectivity strength. Furthermore, we found an association between connectivity strength in a subregion of the forceps minor and the forceps major and clinical variables of importance in the vulnerability to suicidal behavior (anxiety, hopelessness and subjective depressive feelings of depression, respectively).

Trait anxiety linked personality traits have for example previously been associated with a decreased white matter microstructure in the forceps major in healthy subjects, showing that not only the limbic circuitry is of importance in emotion regulation (Westlye et al., 2011).

Due to the general problem of crossing fibers addressed previously, interpretation of the results should be done with caution. Former research has shown that callosal fibers diverging to the cortical endpoint merge with longitudinal projections to that destination (Yeatman et al., 2012). Nonetheless, callosal fibers close to the corpus callosum are tightly bundled and less influenced by crossing fibers, providing support for our interpretation of the results.

Suicidal behavior has been associated with deficits involving decision-making (Richard-Devantoy et al., 2014). The frontal regions in which the network connections associated with the forceps minor were found, have been associated with disadvantages decision-making in suicide attempters (Jollant et al., 2010). While these authors hypothesized that decision-making in suicide attempters is hampered due to dysfunction of the orbitofrontal cortex, our results provide an alternative explanation. It could well be that disadvantages decision-making is caused by a lack of interhemispheric interaction due to a decreased connection strength, which leads to bottom-up attentional control deficits (Banich, 1998). A study by Welcome and Chiarello (2008) provides additional evidence by stating that the interaction between the hemispheres becomes advantages due to bottom-up task related processing demands and not because of top-down knowledge. It could does well be that decision making deficits are present in euthymic suicide attempters when confronted with higher order task demands, due to a lack of interhemispheric connectivity strength, limiting interaction.

In summary, the current findings suggest that a vulnerability to suicidal behavior is associated with a reduced connectivity strength in the minor and the major forceps which coincides with the manifestation of maladaptive clinical characteristics such as hopelessness and subjective feelings of depression, and possibly with cognitive abilities such as difficulties in decision making. Changes in connectivity strength are not related to *5-HTTLPR VNTR* [*rs4795541 (L/S-variant)* and *rs25531* (*L_A_/L_G_/S-variant*)] and *MAOA* polymorphisms. Future longitudinal studies in a larger sample are needed to assess the utility of the demonstrated alterations in predicting the risk of suicide attempts and in serving as a substrate for treatment.

## Author Contributions

SB, EA and CV designed the study, while SB, EA, KT, HV and CV acquired the data. SB, KC, DR and CV analyzed and interpreted the data. SB, KC, DR and CV drafted the manuscript, while EA, KT and HV revised the content. All authors gave written final approval for the article and agreed to be accountable.

## Conflict of Interest Statement

The authors declare that the research was conducted in the absence of any commercial or financial relationships that could be construed as a potential conflict of interest.

## Abbreviations

5-HTTLPR: Serotonin transporter gene promoter
AAL: Automated Anatomic Labeling
BDI-II-NL: Dutch version of the Beck depression inventory
BP: Base pairs
BAS-Fun: BIS/BAS scale Fun Seeking
BAS-Reward: BIS/BAS scale Reward Responsiveness
BAS-Drive: BIS/BAS scale Drive
DWI: Diffusion-weighted imaging volume
EPI: Echo planar imaging
HC: Healthy controls
MAOA: Monoamine oxidase A
MRI: Magnetic Resonance Imaging
NBS: Network Based Statistics
NA: Euthymic outpatients without a history of suicide attempt
SA: Euthymic outpatients with a history of suicide attempt(s)
TE: Echo time
TR: Repetition time
VNTR: Variable number tandem repeat.

## References

[B1] Antypa N. Serretti A. Rujescu D. (2013). Serotonergic genes and suicide: a systematic review. Eur. Neuropsychopharmacol. 23, 1125–1142. 10.1016/j.euroneuro.2013.03.013 23742855

[B2] Bai F. Shu N. Yuan Y. Shi Y. Yu H. Wu D.. (2012). Topologically convergent and divergent structural connectivity patterns between patients with remitted geriatric depression and amnestic mild cognitive impairment. J. Neurosci. 32, 4307–4318. 10.1523/JNEUROSCI.5061-11.2012 22442092 PMC6621223

[B3] Banich M. T. (1998). The missing link: the role of interhemispheric interaction in attentional processing. Brain Cogn. 36, 128–157. 10.1006/brcg.1997.0950 9520311

[B4] Basser P. J. Pajevic S. Pierpaoli C. Duda J. Aldroubi A. (2000a). *In vivo* fiber tractography using DT-MRI data. Magn. Reson. Med 44, 625–632. 10.1002/1522-2594(200010)44:4<625::aid-mrm17>3.0.co;2-o 11025519

[B5] Basser P. J. Pajevic S. Pierpaoli C. Duda J. Aldroubi A. (2000b). *In vivo* fiber tractography using DT-MRI data. Magn. Reson. Med. 44, 625–632. 10.1002/1522-2594(200010)44:4<625::aid-mrm17>3.0.co;2-o 11025519

[B6] Beck A. T. Steer R. A. (1988). Manual for the Beck Hopelessness Scale. San Antonio, TX: Psychological Corporation.

[B7] Beck A. T. Kovacs M. Weissman A. (1979). Assessment of suicidal intention: the scale for suicide ideation. J. Consult. Clin. Psychol. 47, 343–352. 10.1037/0022-006x.47.2.343 469082

[B8] Beck A. T. Ward C. H. Mendelson M. Mock J. Erbaugh J. (1961). An inventory for measuring depression. Arch. Gen. Psychiatry 4, 561–571. 10.1001/archpsyc.1961.01710120031004 13688369

[B9] Beck A. T. Weissman A. Lester D. Trexler L. (1974). The measurement of pessimism: the hopelessness scale. J. Consult. Clin. Psychol. 42, 861–865. 10.1037/h0037562 4436473

[B10] Berlucchi G. Gazzaniga M. S. Rizzolatti G. (1967). Microelectrode analysis of transfer of visual information by the corpus callosum. Arch. Ital. Biol. 105, 583–596. 5585725

[B11] Caeyenberghs K. Leemans A. De Decker C. Heitger M. Drijkoningen D. Linden C. V.. (2012a). Brain connectivity and postural control in young traumatic brain injury patients: a diffusion MRI based network analysis. Neuroimage Clin. 1, 106–115. 10.1016/j.nicl.2012.09.011 24179743 PMC3757722

[B12] Caeyenberghs K. Leemans A. Heitger M. H. Leunissen I. Dhollander T. Sunaert S.. (2012b). Graph analysis of functional brain networks for cognitive control of action in traumatic brain injury. Brain 135, 1293–1307. 10.1093/brain/aws048 22427332

[B13] Caeyenberghs K. Powell H. W. R. Thomas R. H. Brindley L. Church C. Evans J.. (2015). Hyperconnectivity in juvenile myoclonic epilepsy: a network analysis. Neuroimage Clin. 7, 98–104. 10.1016/j.nicl.2014.11.018 25610771 PMC4299970

[B14] Catani M. Thiebaut de Schotten M. (2008). A diffusion tensor imaging tractography atlas for virtual *in vivo* dissections. Cortex 44, 1105–1132. 10.1016/j.cortex.2008.05.004 18619589

[B15] Cyprien F. Courtet P. Malafosse A. Maller J. Meslin C. Bonafé A.. (2011). Suicidal behavior is associated with reduced corpus callosum area. Biol. Psychiatry 70, 320–326. 10.1016/j.biopsych.2011.02.035 21531383

[B16] Desmyter S. Bijttebier S. van Heeringen K. (2013). The role of neuroimaging in our understanding of the suicidal brain. CNS Neurol. Disord. Drug Targets 12, 921–929. 10.2174/18715273113129990093 24040805

[B17] Dougherty R. F. Ben-Shachar M. Bammer R. Brewer A. A. Wandell B. A. (2005). Functional organization of human occipital-callosal fiber tracts. Proc. Natl. Acad. Sci. U S A 102, 7350–7355. 10.1073/pnas.0500003102 15883384 PMC1129102

[B18] Fan Q. Yan X. Wang J. Chen Y. Wang X. Li C.. (2012). Abnormalities of white matter microstructure in unmedicated obsessive-compulsive disorder and changes after medication. PLoS One 7:e35889. 10.1371/journal.pone.0035889 22558258 PMC3338776

[B19] First M. Spitzer R. Gibbon M. Williams J. Benjamin L. (1994). Structured Clinical Interview for DSM-IV Axis II personality disorders (SCID II). New York, NY: Biometric Research Department.

[B20] Fornito A. Yoon J. Zalesky A. Bullmore E. T. Carter C. S. (2011). General and specific functional connectivity disturbances in first-episode schizophrenia during cognitive control performance. Biol. Psychiatry 70, 64–72. 10.1016/j.biopsych.2011.02.019 21514570 PMC4015465

[B21] Franken I. H. A. Muris P. Rassin E. (2005). Psychometric properties of the dutch BIS/BAS scales. J. Psychopathol. Behav. 27, 25–30. 10.1007/s10862-005-3262-2

[B22] Germana C. Kempton M. J. Sarnicola A. Christodoulou T. Haldane M. Hadjulis M.. (2010). The effects of lithium and anticonvulsants on brain structure in bipolar disorder. Acta Psychiatr. Scand. 122, 481–487. 10.1111/j.1600-0447.2010.01582.x 20560901

[B23] Gong G. He Y. Concha L. Lebel C. Gross D. W. Evans A. C.. (2009). Mapping anatomical connectivity patterns of human cerebral cortex using *in vivo* diffusion tensor imaging tractography. Cereb. Cortex 19, 524–536. 10.1093/cercor/bhn102 18567609 PMC2722790

[B24] Hagmann P. Cammoun L. Gigandet X. Meuli R. Honey C. J. Wedeen V. J.. (2008). Mapping the structural core of human cerebral cortex. PLoS Biol. 6:e159. 10.1371/journal.pbio.0060159 18597554 PMC2443193

[B25] Hamilton M. (1960). A rating scale for depression. J. Neurol. Neurosur. Psychiatry 23, 56–62. 10.1136/jnnp.23.1.56 14399272 PMC495331

[B26] Hawton K. van Heeringen K. (2009). Suicide. Lancet 373, 1372–1381. 10.1016/S0140-6736(09)60372-X 19376453

[B27] Hess C. P. Mukherjee P. Han E. T. Xu D. Vigneron D. B. (2006). Q-ball reconstruction of multimodal fiber orientations using the spherical harmonic basis. Magn. Reson. Med. 56, 104–117. 10.1002/mrm.20931 16755539

[B28] Jeurissen B. Leemans A. Jones D. K. Tournier J. D. Sijbers J. (2011). Probabilistic fiber tracking using the residual bootstrap with constrained spherical deconvolution. Hum. Brain Mapp. 32, 461–479. 10.1002/hbm.21032 21319270 PMC6869960

[B29] Jia Z. Huang X. Wu Q. Zhang T. Lui S. Zhang J.. (2010). High-field magnetic resonance imaging of suicidality in patients with major depressive disorder. Am. J. Psychiatry 167, 1381–1390. 10.1176/appi.ajp.2010.09101513 20843871

[B30] Jia Z. Wang Y. Huang X. Kuang W. Wu Q. Lui S.. (2014). Impaired frontothalamic circuitry in suicidal patients with depression revealed by diffusion tensor imaging at 3.0 T. J. Psychiatry Neurosci. 39, 170–177. 10.1503/jpn.130023 24119793 PMC3997602

[B31] Jollant F. Lawrence N. S. Olie E. O’Daly O. Malafosse A. Courtet P.. (2010). Decreased activation of lateral orbitofrontal cortex during risky choices under uncertainty is associated with disadvantageous decision-making and suicidal behavior. Neuroimage 51, 1275–1281. 10.1016/j.neuroimage.2010.03.027 20302946

[B33] Jones D. K. Knösche T. R. Turner R. (2013). White matter integrity, fiber count and other fallacies: the do’s and don’ts of diffusion MRI. Neuroimage 73, 239–254. 10.1016/j.neuroimage.2012.06.081 22846632

[B32] Jones D. K. Leemans A. (2011). Diffusion tensor imaging. Methods Mol. Biol. 711, 127–144. 10.1007/978-1-61737-992-5_6 21279600

[B34] Khalsa S. Mayhew S. D. Chechlacz M. Bagary M. Bagshaw A. P. (2014). The structural and functional connectivity of the posterior cingulate cortex: comparison between deterministic and probabilistic tractography for the investigation of structure-function relationships. Neuroimage 102, 118–127. 10.1016/j.neuroimage.2013.12.022 24365673

[B35] Kim B. Oh J. Kim M. K. Lee S. Tae W. S. Kim C. M.. (2015). White matter alterations are associated with suicide attempt in patients with panic disorder. J. Affect. Disord. 175, 139–146. 10.1016/j.jad.2015.01.001 25617685

[B36] Lebel C. Benner T. Beaulieu C. (2012). Six is enough? Comparison of diffusion parameters measured using six or more diffusion-encoding gradient directions with deterministic tractography. Magn. Reson. Med. 68, 474–483. 10.1002/mrm.23254 22162075

[B37] Leemans A. Jones D. K. (2009). The B-matrix must be rotated when correcting for subject motion in DTI data. Magn. Reson. Med. 61, 1336–1349. 10.1002/mrm.21890 19319973

[B38] Leemans A. Jeurissen B. Sijbers J. Jones D. K. (2009). ExploreDTI: a graphical toolbox for processing, analyzing and visualizing diffusion MR data. 17th Annual Meeting of Intl Soc Mag Reson Med Hawai.

[B39] Lo C. P. Chen S. Y. Chou M. C. Wang C. Y. Lee K. W. Hsueh C. J.. (2007). Diffusion-tensor MR imaging for evaluation of the efficacy of hyperbaric oxygen therapy in patients with delayed neuropsychiatric syndrome caused by carbon monoxide inhalation. Eur. J. Neurol. 14, 777–782. 10.1111/j.1468-1331.2007.01854.x 17594334

[B40] Lopez-Larson M. King J. B. McGlade E. Bueler E. Stoeckel A. Epstein D. J.. (2013). Enlarged thalamic volumes and increased fractional anisotropy in the thalamic radiations in veterans with suicide behaviors. Front. Psychiatry 4:83. 10.3389/fpsyt.2013.00083 23964245 PMC3740266

[B41] Mahon K. Burdick K. E. Wu J. Ardekani B. A. Szeszko P. R. (2012). Relationship between suicidality and impulsivity in bipolar I disorder: a diffusion tensor imaging study. Bipolar Disord 14, 80–89. 10.1111/j.1399-5618.2012.00984.x 22329475 PMC3319758

[B42] Mori S. van Zijl P. C. (2002). Fiber tracking: principles and strategies - a technical review. NMR Biomed. 15, 468–480. 10.1002/nbm.781 12489096

[B43] Mori S. Crain B. J. Chacko V. P. van Zijl P. C. M. (1999). Three-dimensional tracking of axonal projections in the brain by magnetic resonance imaging. Ann. Neurol. 45, 265–269. 10.1002/1531-8249(199902)45:2<265::aid-ana21>3.0.co;2-3 9989633

[B44] Olvet D. M. Peruzzo D. Thapa-Chhetry B. Sublette M. E. Sullivan G. M. Oquendo M. A. (2014). A diffusion tensor imaging study of suicide attempters. J. Psychiatr. Res. 51, 60–67. 10.1016/j.jpsychires.2014.01.002 24462041 PMC4060601

[B45] Overbeek T. Schruers K. Griez E. (1999). Mini International Neuropsychiatric Interview, Dutch version 5.0.0. Maastricht: University of Maastricht.

[B46] Patton J. H. Stanford M. S. Barratt E. S. (1995). Factor structure of the barratt impulsiveness scale. J. Clin. Psychol. 51, 768–774. 10.1002/1097-4679(199511)51:6<768::aid-jclp2270510607>3.0.co;2-1 8778124

[B47] Poudel G. R. Stout J. C. Dominguez D. J. Salmon L. Churchyard A. Chua P.. (2014). White matter connectivity reflects clinical and cognitive status in huntington’s disease. Neurobiol. Dis. 65, 180–187. 10.1016/j.nbd.2014.01.013 24480090

[B48] Ptito M. Leporé F. Lassonde M. Miceli D. Guillemot J. P. (1981). The role of the corpus-callosum and other commissures in the inter-hemispheric transfer of visual information. Rev. Can. Biol. 40, 61–68. 7244318

[B49] Richard-Devantoy S. Berlim M. T. Jollant F. (2014). A meta-analysis of neuropsychological markers of vulnerability to suicidal behavior in mood disorders. Psychol. Med. 44, 1663–1673. 10.1017/s0033291713002304 24016405

[B50] Rubinov M. Bassett D. S. (2011). Emerging evidence of connectomic abnormalities in schizophrenia. J. Neurosci. 31, 6263–6265. 10.1523/JNEUROSCI.0382-11.2011 21525265 PMC6622649

[B51] Schmand B. Lindeboom J. van Harskamp F. (1992). “Nederlandse leestest voor volwassenen,” in Neuropsychologische Diagnostiek eds Bouma A. Mulder J. Lindeboom J. (Lisse: Swets and Zeitlinger).

[B52] Skudlarski P. Jagannathan K. Calhoun V. D. Hampson M. Skudlarska B. A. Pearlson G. (2008). Measuring brain connectivity: diffusion tensor imaging validates resting state temporal correlations. Neuroimage 43, 554–561. 10.1016/j.neuroimage.2008.07.063 18771736 PMC4361080

[B53] Tournier J. D. Mori S. Leemans A. (2011). Diffusion tensor imaging and beyond. Magn. Reson. Med. 65, 1532–1556. 10.1002/mrm.22924 21469191 PMC3366862

[B54] Tzourio-Mazoyer N. Landeau B. Papathanassiou D. Crivello F. Etard O. Delcroix N.. (2002). Automated anatomical labeling of activations in SPM using a macroscopic anatomical parcellation of the MNI MRI single-subject brain. Neuroimage 15, 273–289. 10.1006/nimg.2001.0978 11771995

[B55] van der Ploeg H. M. (2000). Handleiding bij de Zelf-beoordelings vragenlijst ZBV: Een Nederlandstalige bewerking van Spielberger State-trait anxiety inventory STAI-DY. Lisse: Swets Test Publishers.

[B56] van Heeringen C. Bijttebier S. Godfrin K. (2011). Suicidal brains: a review of functional and structural brain studies in association with suicidal behavior. Neurosci. Biobehav. Rev. 35, 688–698. 10.1016/j.neubiorev.2010.08.007 20826179

[B58] van Heeringen K. Bijttebier S. Desmyter S. Vervaet M. Baeken C. (2014). Is there a neuroanatomical basis of the vulnerability to suicidal behavior? A coordinate-based meta-analysis of structural and functional MRI studies. Front. Hum. Neurosci. 8:824. 10.3389/fnhum.2014.00824 25374525 PMC4205829

[B57] van Heeringen K. Mann J. J. (2014). The neurobiology of suicide. Lancet Psychiatry 1, 63–72. 10.1016/S2215-0366(14)70220-2 26360403

[B59] Vernon A. C. Natesan S. Crum W. R. Cooper J. D. Modo M. Williams S. C.. (2012). Contrasting effects of haloperidol and lithium on rodent brain structure: a magnetic resonance imaging study with postmortem confirmation. Biol. Psychiatry 71, 855–863. 10.1016/j.biopsych.2011.12.004 22244831

[B60] Wedeen V. J. Wang R. P. Schmahmann J. D. Benner T. Tseng W. Y. Dai G.. (2008). Diffusion spectrum magnetic resonance imaging (DSI) tractography of crossing fibers. Neuroimage 41, 1267–1277. 10.1016/j.neuroimage.2008.03.036 18495497

[B61] Welcome S. E. Chiarello C. (2008). How dynamic is interhemispheric interaction? Effects of task switching on the across-hemisphere advantage. Brain Cogn. 67, 69–75. 10.1016/j.bandc.2007.11.005 18206285 PMC2486493

[B62] Westlye L. T. Bjornebekk A. Grydeland H. Fjell A. M. Walhovd K. B. (2011). Linking an anxiety-related personality trait to brain white matter microstructure: diffusion tensor imaging and harm avoidance. Arch. Gen. Psychiatry 68, 369–377. 10.1001/archgenpsychiatry.2011.24 21464361

[B63] WHO (2014). Preventing Suicide: A Global Imperative. Geneva: World Health Organization.

[B64] Yamamoto A. Miki Y. Urayama S. Fushimi Y. Okada T. Hanakawa T.. (2007). Diffusion tensor fiber tractography of the optic radiation: analysis with 6-, 12-, 40- and 81-directional motion-probing gradients, a preliminary study. Am. J. Neuroradiol. 28, 92–96. 17213432 PMC8134109

[B65] Yeatman J. D. Dougherty R. F. Myall N. J. Wandell B. A. Feldman H. M. (2012). Tract profiles of white matter properties: automating fiber-tract quantification. PLoS One 7:e49790. 10.1371/journal.pone.0049790 23166771 PMC3498174

[B66] Yurgelun-Todd D. A. Bueler C. E. McGlade E. C. Churchwell J. C. Brenner L. A. Lopez-Larson M. P. (2011). Neuroimaging correlates of traumatic brain injury and suicidal behavior. J. Head Trauma Rehabil. 26, 276–289. 10.1097/htr.0b013e31822251dc 21734511

[B67] Zalesky A. Fornito A. Bullmore E. T. (2010). Network-based statistic: identifying differences in brain networks. Neuroimage 53, 1197–1207. 10.1016/j.neuroimage.2010.06.041 20600983

[B68] Zalesky A. Fornito A. Seal M. L. Cocchi L. Westin C. F. Bullmore E. T.. (2011). Disrupted axonal fiber connectivity in schizophrenia. Biol. Psychiatry 69, 80–89. 10.1016/j.biopsych.2010.08.022 21035793 PMC4881385

[B69] Zimmerman M. Martinez J. H. Young D. Chelminski I. Dalrymple K. (2013). Severity classification on the hamilton depression rating scale. J. Affect. Disord. 150, 384–388. 10.1016/j.jad.2013.04.028 23759278

