# Supplementary material for: The Vulnerability to Suicidal Behavior is Associated with Reduced Connectivity Strength
Source: Front Hum Neurosci. 2015 Nov 30;9:632. doi: 10.3389/fnhum.2015.00632 (PMC4663245; doi:10.3389/fnhum.2015.00632)
Supplement: Supplementary file 1 [file Table_1.docx]

|  | **Connections** | **Post-hoc contrasts**  **(Mann–Whitney U tests)** |
| --- | --- | --- |
| **Number of streamline** |  |  |
|  | Frontal_Mid_R →  Frontal_Mid_Orb_R | HC>NA (U = 63.5, p < 0.05 );  HC=SA (U = 72.5, p = 0.11);  NA=SA (U = 85.5, p = 0.57) |
|  | Olfactory_L →  Frontal_Sup_Medial_L | HC=NA (U = 77.0, p = 0.54);  HC=SA (U = 88.0, p = 0.33);  NA>SA (U = 47.5, p < 0.05) |
|  | Olfactory_R →  Frontal_Sup_Medial_L | HC<NA (U = 74.5, p < 0.05 );  HC=SA (U =102.0, p = 0.72);  NA=SA (U = 65.0, p = 0.13) |
|  | Olfactory_L →  Frontal_Sup_Medial_R | HC=NA (U = 82.0, p = 0.08);  HC=SA (U = 84.0, p = 0.27);  NA>SA (U = 54.5, p < 0.05) |
|  | Olfactory_R →  Frontal_Sup_Medial_R | HC=NA (U = 76.0, p = 0.05);  HC=SA (U = 108.5, p = 0.93);  NA=SA (U = 58.5, p = 0.07 ) |
|  | Frontal_Sup_Orb_L →  Frontal_Med_Orb_L | HC<NA (U = 63.0, p < 0.05 );  HC<SA (U = 54.0, p < 0.05);  NA=SA (U = 73.5, p = 0.27 ) |
|  | Frontal_Med_Orb_R →  Rectus_L | HC>NA (U = 66.5, p < 0.05);  HC>SA (U = 37.5, p < 0.01);  NA=SA (U = 81.5, p = 0.46 ) |
|  | Frontal_Sup_Orb_L →  Amygdala_L | HC<NA (U = 69.0, p < 0.05);  HC<SA (U = 58.5, p < 0.05);  NA=SA (U = 79.0, p = 0.39 ) |
|  | Rectus_L →  Amygdala_L | HC>NA (U = 65.0, p < 0.05);  HC=SA (U = 81.0, p = 0.20);  NA=SA (U = 72.0, p = 0.24) |
|  | ParaHippocampal_R →  Cuneus_L | HC>NA (U = 45.0, p < 0.01);  HC>SA (U = 60.5, p < 0.05);  NA=SA (U = 65.5, p = 0.07 ) |
|  | Frontal_Med_Orb_L →  Lingual_L | HC<NA (U = 49.5, p < 0.01);  HC=SA (U = 85.5, p = 0.13);  NA=SA (U = 63.5, p = 0.09) |
|  | Cuneus_L →  Lingual_R | HC>NA (U = 49.5, p < 0.01);  HC>SA (U = 39.0, p < 0.01);  NA=SA (U = 93.5, p = 0.85) |
|  | ParaHippocampal_R →  Occipital_Sup_L | HC>NA (U = 34.5, p < 0.001);  HC>SA (U = 53.5, p < 0.05);  NA=SA (U = 73.0, p = 0.09) |
|  | Olfactory_L →  Occipital_Mid_L | HC>NA (U = 65.0, p < 0.05);  HC>SA (U = 49.5, p < 0.05);  NA=SA (U = 87.5, p = 0.64) |
|  | Frontal_Med_Orb_L →  Occipital_Mid_L | HC<NA (U = 41,5., p < 0.01);  HC=SA (U = 82.0, p = 0.16);  NA>SA (U = 51.5, p < 0.05 ) |
|  | Frontal_Sup_L →  Parietal_Sup_L | HC=NA (U = 90.5, p = 0.15);  HC=SA (U = 103.5, p = 0.76);  NA=SA (U = 65.5, p = 0.13) |
|  | Hippocampus_R →  Parietal_Sup_L | HC>NA (U = 65.0, p < 0.05);  HC=SA (U = 65.0, p = 0.05);  NA=SA (U =83.0, p = 0.49) |
|  | Calcarine_R →  Parietal_Sup_L | HC=NA (U = 90.0, p = 0.14);  HC>SA (U = 61.0, p < 0.05);  NA=SA (U = 74.0, p = 0.21) |
|  | Parietal_Sup_L →  Paracentral_Lobule_L | HC<NA (U = 56.0, p < 0.01);  HC<SA (U = 50.5, p < 0.05);  NA=SA (U = 56.5, p = 0.06) |
|  | Precuneus_L →  Paracentral_Lobule_L | HC<NA (U = 67.5, p < 0.05);  HC=SA (U = 92.5, p = 0.45);  NA=SA (U =57.0, p = 0.06) |
|  | Precuneus_L →  Paracentral_Lobule_R | HC<NA (U = 65.0, p < 0.05);  HC=SA (U = 71.0, p = 0.10);  NA=SA (U = 78.5, p = 0.38) |
|  | Frontal_Sup_Orb_L →  Caudate_L | HC>NA (U = 61.5, p < 0.05);  HC>SA (U = 61.0, p < 0.05);  NA=SA (U = 88.5, p = 0.68) |
|  | Frontal_Sup_Medial_L →  Caudate_L | HC<NA (U = 63.0, p < 0.05);  HC=SA (U = 95.5, p = 0.52);  NA>SA (U = 34.0, p < 0.01 ) |
|  | Frontal_Sup_Orb_R →  Caudate_R | HC>NA (U = 68.0, p < 0.05);  HC>SA (U = 49.5, p < 0.05);  NA=SA (U = 97.0, p = 0.98) |
|  | Frontal_Mid_Orb_R →  Caudate_R | HC>NA (U = 45.5, p < 0.01);  HC>SA (U = 54.0, p < 0.05);  NA=SA (U = 60.5, p = 0.09) |
|  | Frontal_Med_Orb_L →  Putamen_L | HC<NA (U = 45.5, p < 0.01);  HC=SA (U = 72.0, p = 0.08);  NA=SA (U = 56.5, p = 0.06) |
|  | Frontal_Sup_Orb_R →  Putamen_R | HC>NA (U = 75.0, p < 0.05);  HC>SA (U = 38.5, p < 0.01);  NA>SA (U = 63.0, p = 0.11) |
|  | Frontal_Med_Orb_R →  Putamen_R | HC<NA (U = 85.0, p < 0.05);  HC=SA (U = 110.5, p = 1.00);  NA>SA (U = 65.0, p < 0.05 ) |
|  | Calcarine_L →  Pallidum_L | HC=NA (U = 104.5, p = 0.38);  HC<SA (U = 58.0, p < 0.05 );  NA=SA (U = 61.5 , p = 0.10) |
|  | Calcarine_R →  Pallidum_L | HC=NA (U = 123.5, p = 0.81);  HC=SA (U = 80.0, p = 0.10);  NA=SA (U = 68.0 , p = 0.07) |
|  | Lingual_L →  Pallidum_L | HC=NA (U = 119.5, p = 0.76);  HC<SA (U = 63.0, p < 0.05 );  NA<SA (U = 60.0 , p = 0.08) |
|  | Frontal_Med_Orb_L →  Temporal_Mid_L | HC<NA (U = 66.0, p < 0.01);  HC=SA (U = 98.5, p = 0.52);  NA=SA (U = 64.5 , p = 0.10) |

Supplementary table 1. Post-Hoc contrasts not showing a significant difference between suicide attempters compared to non-attempters and healthy controls.
